# Novel Circo-Like Virus Detected in a Croatian Bat Population

**DOI:** 10.1128/MRA.00280-19

**Published:** 2019-04-18

**Authors:** Ivana Šimić, Tomaž Mark Zorec, Nina Krešić, Mario Poljak, Tomislav Bedeković, Ivana Lojkić

**Affiliations:** a Department of Virology, Croatian Veterinary Institute, Zagreb, Croatia; b Institute of Microbiology and Immunology, Faculty of Medicine, University of Ljubljana, Ljubljana, Slovenia; Queens College

## Abstract

Viral metagenomics analysis of samples from bats has been carried out as part of bat rabies surveillance in Croatia. Here, we report the complete genome sequence of a novel circo-like virus isolated from a sample of Miniopterus schreibersii bat guano determined by Illumina next-generation sequencing.

## ANNOUNCEMENT

The family *Circoviridae* includes two genera, *Circovirus* and *Cyclovirus*, whose circular single-stranded DNA (ssDNA) molecules encode at least two major open reading frames (ORFs), the replication-associated (Rep) and the capsid protein. Members of the *Circoviridae* have been detected in numerous vertebrate and invertebrate organisms (1). Here, we report the complete genome sequence of a novel circo-like virus, Croatia 17_S17, isolated from guano of the Miniopterus schreibersii bat, determined by next-generation sequencing.

Nucleic acids were extracted using the iPrep virus kit (Invitrogen) according to the manufacturer’s instructions. A sequencing library was constructed using the Nextera XT DNA library preparation kit and sequenced using a MiSeq reagent kit v2 in paired-end mode (2 × 250 nucleotides [nt]).

The complete genome sequence (length, 2,440 bp; GC content, 38%; average depth of coverage, 38.84×) of circo-like virus Croatia 17_S17 was identified among sequence contigs (assembled *de novo* using SPAdes v3.12.0 [2] using the complete raw read data set and default assembler parameters) using DIAMOND blastx (3) (version, GitHub commit c335eda162cee51dadc3209e1c0e5241b493fb61) by mapping against the viral (taxid 10239) nonredundant protein data set, obtained from NCBI (11 June 2018), at the cutoff E value of 10^−4^ and NCBI blastx (https://blast.ncbi.nlm.nih.gov/Blast.cgi). The sequence indicated the presence of a *Circoviridae* Rep gene. The completeness and circularity of the sequence were determined by identification of matching 5′ and 3′ ends, and moreover, the sequence indicated the presence of a cyclovirus-like nonanucleotide motif (TAATACTAT) flanked by a pseudopalindromic sequence representing the stem-loop (4). The sequence residues at the 3′ end (matching the 5′ end) were manually trimmed, and the sequence was rotated to start with the stem-loop. Remapping to the novel genome sequence using the BWA v0.7.17-r1188 with default parameters and screening with Pilon v1.22 (5) (with the parameter “–fix all”) did not show evidence of misassembly. The average depth of coverage of the novel genome sequence was calculated on the basis of read remapping using SAMtools v1.3 and Awk v4.1.4 using the following command: “samtools depth -a <bam file> | awk ‘{sum+=$3} END {print sum/NR}’.”

The novel genome sequence contains four ORFs (identified using ORFfinder; parameters “-s 0 -ml 300”) encoding four putative proteins, including a putative Rep, and three hypothetical proteins (predicted by annotation transfer according to NCBI blastp similarity). The Rep protein was examined for the presence of conserved amino acid motifs, characteristic of circular ssDNA viruses, and showed the presence of all amino acid motifs, characteristic of *Circoviridae* replication-associated proteins. It has three conserved rolling circle replication (RCR) motifs in the N-terminal region, RCR motif I (FTEFN), RCR motif II (VHVQG), and RCR motif III (YCKK), as well as superfamily 3 helicase Walker A, B, and C motifs (1). The RCR motif I does not unambiguously correspond to those found in circoviruses or cycloviruses but was identified as described by Castrignano et al. (6). The RCR motif II, VHVQG, is closer to the described nanovirus xHUQG motif (“U” represents bulky hydrophobic amino acids—I, L, V, M, F, Y, W). The RCR motif III, YCKK, is compatible with the Rep proteins of circovirus, cyclovirus, geminivirus, and nanovirus. Moreover, the helicase superfamily 3 Walker A, B, and C motifs could be identified in the C-terminal region (GPPGTGKS, IIDDF, ITSN) (1).

The virus reported here exhibits a type V circular ssDNA virus genome organization (1).

**FIG 1:**
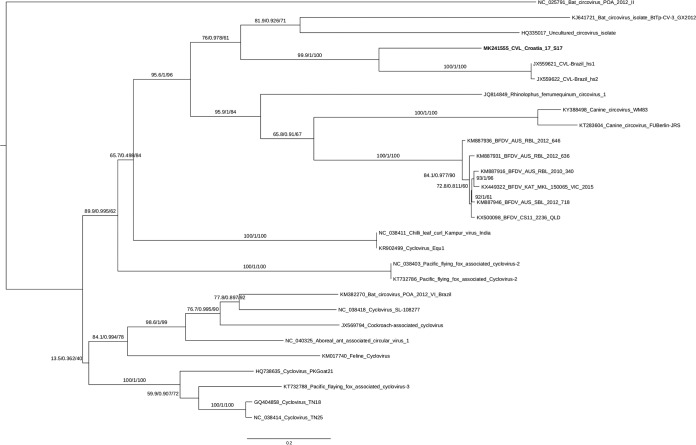
Phylogenetic tree of circo- and circo-like virus Rep sequences from diverse species and samples. The maximum likelihood phylogenetic tree was generated with the TIM3+F+R3 substitution model using IQ-TREE (parameters “-alrt 1000 -abayes –bb 1000”). The sequence identifiers include the NCBI accession number and isolate name. The novel Croatian isolate (Circo-like virus Croatia 17_S17) is indicated in bold. Branch supports are provided in the following format: SH-aLRT (%)/aBayes/ultrafast bootstrap (%).

Phylogenetic analysis conducted using IQ-TREE (7–9) (parameters “-alrt 1000 -abayes -bb 1000”) (Fig. 1), based on the multiple sequence alignment of the Rep proteins (aligned using Muscle v3.8.31), demonstrated that the circo-like virus Croatia 17_S17 clusters close to circo-like virus sequences from Brazil, CLV-BR hs1 and CLV-BR hs2 (GenBank accession no. JX559621 and JX559622), which were detected in human feces (6, 10).

### Data availability.

The complete genome sequence of circo-like virus Croatia 17_S17 has been deposited at GenBank under the accession no. MK241555. The raw reads were deposited in SRA under the accession no. SRR8759221.
